# Bio301: A Web-Based EST Annotation Pipeline That Facilitates Functional Comparison Studies

**DOI:** 10.5402/2012/139842

**Published:** 2011-09-27

**Authors:** Yen-Chen Chen, Yun-Ching Chen, Wen-Dar Lin, Chung-Der Hsiao, Hung-Wen Chiu, Jan-Ming Ho

**Affiliations:** ^1^Institute of Information Science, Academia Sinica, 128 Academia Road, Section 2, Nankang, Taipei 115, Taiwan; ^2^Department of Biomedical Engineering, The Whitaker Biomedical Engineering Institute at Johns Hopkins University School of Medicine, 720 Rutland Avenue, Baltimore, MD 21205, USA; ^3^Institute of Plant and Microbial Biology, Academia Sinica, 128 Academia Road, Section 2, Nankang, Taipei 115, Taiwan; ^4^Department of Bioscience Technology, Chung Yuan Christian University, 200 Chung Pei Road, Chung Li City 32073, Taiwan; ^5^Graduate Institute of Biomedical Informatics, Taipei Medical University, 250 Wu-Hsing Street, Taipei City 110, Taiwan

## Abstract

In this postgenomic era, a huge volume of information derived from expressed sequence tags (ESTs) has been constructed for functional description of gene expression profiles. Comparative studies have become more and more important to researchers of biology. In order to facilitate these comparative studies, we have constructed a user-friendly EST annotation pipeline with comparison tools on an integrated EST service website, Bio301. Bio301 includes regular EST preprocessing, BLAST similarity search, gene ontology (GO) annotation, statistics reporting, a graphical GO browsing interface, and microarray probe selection tools. In addition, Bio301 is equipped with statistical library comparison functions using multiple EST libraries based on GO annotations for mining meaningful biological information.

## 1. Motivation

Expressed sequence tags (ESTs) [1] are small pieces of DNA sequences (usually 200 to 500 nucleotides long) derived by either unidirectional or bidirectional sequencing of cDNA libraries. The information generated from ESTs has been utilized not only to identify novel gene transcripts, gene locations, and intron-exon boundaries in human and mouse genome drafts [2, 3] but also to assess gene expression levels of given tissues [4].

The large volume of information generated by the rapidly increasing number of ESTs—59 million EST entries in the dbEST in January 2009 alone—provides an excellent resource for comparative studies, so we have constructed an EST service website, Bio301, to facilitate comparative studies based on these EST data. Bio301 is equipped with not only an EST annotation pipeline but also functional comparative functionality. Bio301 has five characteristics considered to be essential for EST analysis tools that aid in functional comparative studies: accurate preprocessing, advanced functional annotation methods, flexibility in comparing multiple EST libraries, retrieval of EST data with respect to the annotation ontology, and integrated online EST service open to the entire research community.

First, Bio301 preprocesses ESTs accurately by cleaning, clustering, and assembling them. These tasks are very important because accurate preprocessing leads to accurate functional annotation, which is crucial for functional comparison studies. Bio301 uses one of the best programs for sequence cleaning, SeqClean (http://compbio.dfci.harvard.edu/tgi/software/). Concordantly, Bio301 also uses state-of-the-art programs for clustering and assembly, TGICL and CAP3 [5, 6]. Since reference genomes with extensive genome annotation have been shown to be helpful for annotation and clustering [7, 8], Bio301 also is equipped with an option for clustering ESTs wherein ESTs are mapped into a user-specified genome using BLAT [9], and Bio301 then assembles these ESTs using CAP3. Bio301 also has an option for processing input data in ACE format, a format commonly used by many third-party assembly programs or next-generation sequencing technologies like the Roche 454 pipeline.

Second, Bio301 has advanced functional annotation methods. These methods, in conjunction with public sequence database searching, enable biologists to deal with uncommon organisms. Bio301 utilizes E2D [10] to retrieve GO annotations via recognized potential InterPro [11] domains. In testing, E2D performed as well as the InterProScan (precision 96.1% versus 96.6 and recall 53.8% versus 30.3%) but ran approximately 69 times faster [10]. In our case study (see Section 3), the combination of E2D and public sequence database searching allowed us to annotate 4% ~ 6% more tentative unique genes than we were able to do previously with public sequence database searching alone.

Third, Bio301 gives users flexibility in comparing multiple EST libraries by performing statistical comparisons based on expression profiles and GO annotations. This flexibility in comparing multiple EST libraries is essential because EST libraries are often sampled from organisms that are of special interest to biologists. Biologists often have to sequence more than one library in order to determine the difference in genomic function. However, they often encounter problems when trying to functionally compare two or more EST libraries, especially when the annotations of those EST libraries were not derived using the same vocabulary. Bio301 first performs statistical comparisons based on expression profiles and GO annotations, and it then ranks GO terms according to significant levels computed by the Chi-squared test for goodness of fit [12]. For example, a top-ranked GO term implies a stronger expressional difference between libraries with respect to this function.

Fourth, Bio301 enables users to retrieve EST data related to its annotation ontology. This kind of data aids biologists in conducting further investigation of specific EST data. As shown in the Table 1, users can select EST data that are related to a given GO term belonging to a given library. This feature helps biologists identify key transcripts under specified conditions.

Last, Bio301 integrates EST services and makes them easily accessible online to the entire research community. Bio301 should not only eliminate some dependence on in-house IT support, but, through Bio301's library management interface, it should make it easier for researchers to share EST libraries with authorized users and to compare these EST libraries in a kind of next-generation collaboration over the Internet.

In the creation of our next-generation Bio301, we analyzed various advanced EST analysis tools that have been developed in recent years and looked for their best features. For example, annot8r [13], ESTpiper [14], and EST2uni [15] have extensive annotation modules. GO-Diff [16] mines functional differences between two annotated EST libraries. OREST [7] has a user-friendly web interface for annotating ESTs and compares them with model organisms. Since each of these tools has its own strength and shortcomings, in the creation of Bio301, we sought to combine the best aforementioned features of all of the aforementioned advanced EST tools such that Bio301 would facilitate ongoing and future functional comparison studies based on EST data.

## 2. Comparison Methods

EST libraries are often sampled from organisms that are of special interest to biologists. For instance, biologists may sequence more than one library from different tissues of the same organism or from similar tissues belonging to different organisms in order to study the difference in genomic functions. However, biologists often encounter problems when trying to compare two or more EST libraries. These problems occur for two reasons. First, the numbers of ESTs in different libraries are not normalized. Second, the annotations of EST data in different libraries may not be derived using the same vocabulary. Developing a unified approach for library comparison is thus very important if the research community wishes to achieve the aforementioned goal of comparing multiple EST libraries. In Bio301, we overcame these constraints by designing two library comparison approaches based on GO terms.

Initially, an *expression matrix* is constructed in which the rows are indexed by GO terms and the columns are indexed by libraries. For convenience, let *N* denote the number of libraries and *M* denote the number of involved GO terms. Each cell of the matrix is filled with *f*_*ij*_, the number of ESTs that belong to the corresponding library (column *i*) and GO term (row *j*).

Our first approach considers the hierarchical clustering of libraries based on the expression patterns of libraries, that is, the hierarchical clustering of *N* vectors (*f*_*i*1_′, *f*_*i*2_′,…, *f*_*iM*_′) for *i* = 1,2,…, *N*, where

(1) $$f_{ij}^{\prime }=f_{ij}÷\overset{M}{\underset{y=1}{\sum }}f_{iy}\times 1000,$$

**Figure 1:**
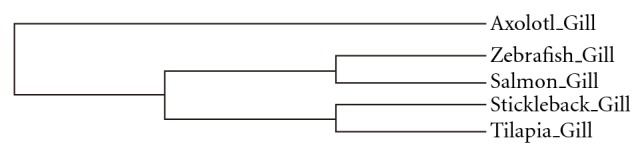
The axolotl gill is not included in the family of fish gills.

*f*_*ij*_′ here expresses the average number of ESTs that belong to GO term *j* for every 1000 ESTs from library *i*. Through this hierarchical clustering, the libraries are clustered in such a way that those with similar expression patterns (with respect to GO terms) are “closer” to each other than to libraries with dissimilar patterns. The results are presented as a hierarchical cluster tree, as shown in Figure 1.

While the first approach clusters normalized columns in the *expression matrix*, the second comparison ranks rows according to their relative deviation from an expected frequency. By treating each row *j* as a vector of frequencies (*f*_1*j*_, *f*_2*j*_,…, *f*_*Nj*_), the expression frequencies of libraries with respect to GO term *j*, each row *j* is assigned a Chi-squared value *χ*_*j*_^2^ according to the following formula:

(2) $$\chi_{j}^{2}=\overset{N}{\underset{x=1}{\sum }}\frac{{\left(f_{xj}-e_{xj}\right)}^{2}}{e_{xj}},$$

where (1) *e*_1*j*_, *e*_2*j*_,…, *e*_*Nj*_ are proportional to the numbers of ESTs in libraries 1,2,…, *N* and (2) ∑_*x*=1_^*N*^ *e*_*xj*_ = ∑_*x*=1_^*N*^ *f*_*xj*_. This formula is the same as the one that is used to calculate the Chi-squared value for goodness of fit [12]. A higher *χ*_*j*_^2^ value means that at least one library has a stronger difference between its empirical expression level and the expected expression level. On the comparison result page, GO terms are ranked according to the Chi-squared values, from high to low. That is, GO terms with stronger deviation in expression from the expectation have higher ranks. As in the conventional Chi-squared test, we compute the *P* values for all related GO terms. However, it should be noted that first, the Chi-squared test might not be appropriate if some of the expected frequencies are too small [12]. Second, the *P* values might be too significant if the total number of ESTs is large [17]. That is, users should treat the ranks and *P* values as references, not as sufficient evidence *per se*. GO IDs and GO terms are shown in the attached ranked *expression matrix* (see Table 1). Users may click on the GO IDs to obtain term descriptions from the AmiGO website (http://www.genedb.org/cgi-bin/amigo/go.cgi/ ) or click on the number of ESTs to obtain the corresponding TUGs. Although this comparison is similar to the one provided by GO-Diff [16], our method allows for the comparison of more than two libraries simultaneously and, furthermore, it is integrated well into the Bio301 website.

## 3. Case Study

One of our major motivations in developing Bio301 was to enable users like ourselves to compare the genomic functions of different cDNA libraries derived from a single or multiple species. In the development of Bio301, we used a particular case study to assess its capacity to carry out this task. Our case study focused on the difference between the genomic functions of the gills of fishes and those of amphibian species at the molecular level, of which currently much still remains unknown. In aquatic lower vertebrates, for example, fish, gills are believed to play an essential role in both osmoregulation and respiration [18]. However, in most amphibian species, gills are merely transient structures and are thought to play a more important role in respiration than in osmoregulation. With the aid of Bio301, we sought an answer to the question: how much of a difference is there between the genomic functions of gills in aquatic lower vertebrates and amphibians at the molecular level? 

To address this question, we first constructed gill cDNA libraries of zebrafish and tilapia. We also collected gill ESTs from salmon [19], stickleback (from NCBI dbEST (http://www.ncbi.nlm.nih.gov/dbEST/)), and axolotl [20] for cross-species comparison. In Supplementary Material available online at doi: 10.1155/2012/139842, we summarize the information about EST sequencing, clustering, and annotation. With the aid of Bio301, we were able to compare the libraries and obtained the following results. First, the functional hierarchical clustering showed that the axolotl gill is not a member of the fish gill family (Figure 1). Second, respiration-related GO terms, for example, “hemoglobin complex,” “oxygen binding,” and “heme binding,” were ranked at the top because of their higher expression levels in axolotl gills rather than in fish species Table 1. Last, the discovery rates of osmoregulation-related GO terms like “sodium:potassium-exchanging ATPase activity” did not show significant deviation in expression (data not shown). The first and second results both follow the prevailing understanding of biology, but the second result confirms what many biologists have speculated, namely, that the axolotl gill does play a more important role in respiration at the molecular level. The EST libraries, being comprised of samples in steady states, that is, samples with less osmoregulation, might account for the lack of significant deviation in expression in the third observation.

## 4. Concluding Remarks

Compared to other existing EST analysis tools, Bio301 has the following advantages. First, it combines advanced functional annotation methods and thus enables users to annotate more genes than they could with one method alone (see Supplementary Material), which is particularly useful for studying uncommon organisms. Second, its library comparison module gives users the flexibility of comparing any number of libraries at the same time using statistical methods. Third, the well-designed web interface does not require switching among different bioinformatics tools and can be accessed using any operating system platform without any in-house IT support. Our case study shows that this combination of features yields meaningful biological information and ideas for further investigation.

Although, in this paper, we emphasized Bio301's effectiveness in functional comparative studies, Bio301 could also be effective in at least other applications: (1) preprocessing and/or annotation of nucleotide sequences, (2) comparative studies on in-house EST libraries and/or publicly available EST libraries, for example, libraries in the dbEST database, and (3) design of cDNA/oligo array probes for an EST dataset. Additionally, the ACE input option also enables all aforementioned functionalities for preassembled data from third-party software or next-generation sequencing technologies, like the Roche 454 pipeline.

## Supplementary Material



## Figures and Tables

**Table 1:** Respiration-related GO terms are ranked at the top. The numbers in the second (tilapia) to sixth (zebrafish) columns are normalized to an average number of ESTs per 1000 ESTs from corresponding library. This normalization helps users understand the reason why certain GO terms are ranked at the top. In this table, respiration-related GO terms are ranked at the top because the axolotl gill has higher expression levels associated with these GO terms than the fish does.

Gene ontology ID	Tilapia_Gill no. of reads	Axolotl_Gill no. of reads	Salmon_Gill no. of reads	Stickleback_Gill no. of reads	Zebrafish_Gills no. of reads	Chi-square test	*P* value	Gene ontology terms	Classification
GO:0005833	1	**298**	74	0	85	10711.06	0	Hemoglobin complex	C
GO:0019825	9	**298**	74	0	85	10256.62	0	Oxygen binding	F
GO:0015671	9	**298**	74	0	85	10246.30	0	Oxygen transport	P
GO:0015669	9	**298**	74	0	85	10236.92	0	Gas transport	P
GO:0005344	9	**298**	74	0	85	10230.22	0	Oxygen transporter activity	F
GO:0044445	25	**314**	88	4	126	8748.77	0	Cytosolic part	C
GO:0005840	97	**74**	174	6	249	7440.77	0	Ribosome	C
GO:0020037	24	**301**	77	7	89	7292.60	0	Heme binding	F
GO:0046906	24	**301**	77	7	89	7292.60	0	Tetrapyrrole binding	F
GO:0003735	95	**72**	166	7	232	6607.10	0	Structural constituent of ribosome	F
GO:0030529	107	82	185	15	255	6033.01	0	Ribonucleoprotein complex	C
GO:0005829	45	326	107	20	150	5362.74	0	Cytosol	C
GO:0032991	168	455	317	83	397	5190.18	0	Macromolecular complex	C
GO:0005506	37	321	89	24	106	4427.26	0	Iron ion binding	F
GO:0001666	0	2	1	1	84	3853.64	0	Response to hypoxia	P
GO:0044444	237	465	373	149	476	3187.15	0	Cytoplasmic part	C
GO:0030097	3	0	1	2	87	3143.89	0	Hemopoiesis	P
GO:0048534	4	0	1	2	87	3068.34	0	Hemopoietic or lymphoid organ development	P
GO:0043228	139	143	219	57	294	2994.55	0	Non-membrane-bounded organelle	C
GO:0043232	139	143	219	57	294	2994.55	0	Intracellular non-membrane-bounded organelle	C
GO:0002520	4	0	1	2	87	2987.65	0	Immune system development	P
GO:0022892	57	319	105	42	143	2885.54	0	Substrate-specific transporter activity	F
GO:0005215	63	322	117	50	162	2543.00	0	Transporter activity	F
GO:0006412	118	87	178	47	246	2494.78	0	Translation	P
