# Supplementary material for: Bio301: A Web-Based EST Annotation Pipeline That Facilitates Functional Comparison Studies
Source: ISRN Bioinform. 2011 Sep 27;2012:139842. doi: 10.5402/2012/139842 (PMC4407203; doi:10.5402/2012/139842)
Supplement: Supplementary file 1 [file 139842.f1.doc]

# Supplementary Data 2: Comparison of expressed sequence tags generated from gill cDNA libraries

^1^ Redundancy = 1−(No. of TUGs / EST analyzed) × 100%

^2^ RefSeq RNA hits = number of TUGs without any hits in the UniProt database that have hits in RefSeq mRNA database

^3^ E2D for 'no hit' TUGs = number of TUGs without any hits in the public database that were annotated by E2D

|  | | Tilapia  *Oreochromis*  *mossambicus* | Zebrafish  *Danio*  *rerio* | Salmon  *Salmo*  *salar* | Stickleback  *Gasterosteus*  *aculeatus* | Axolotl  *Ambystoma*  *mexicanum* |
| --- | --- | --- | --- | --- | --- | --- |
| EST reads | | 5317 | 3028 | 4017 | 50272 | 1942 |
| EST analyzed | | 5309 | 3026 | 3969 | 50217 | 1935 |
| TUGs | | 3514 | 1400 | 2112 | 27137 | 1344 |
| Library redundancy^1^ | | 33% | 54% | 47% | 46% | 31% |
| TUSs | | 3025 | 1145 | 1706 | 24020 | 1190 |
| TUCs | | 489 | 255 | 406 | 3117 | 154 |
| T UG | Full-length TUGs | 282 (6%) | 1106 (79%) | 383 (18%) | 4214 (16%) | 710 (53%) |
|  | Uniprot hits | 1924 (55%) | 1275 (91%) | 1247 (59%) | 15393 (57%) | 970 (72%) |
|  | RefSeq RNA hits^2^ | 251 (7%) | 76 (5%) | 295 (14%) | 2278 (8%) | 29 (5%) |
|  | No hit | 1339 (38%) | 49 (4%) | 570 (27%) | 9466 (35%) | 345 (26%) |
|  | E2D for 'no hit' TUGs^3^ | 141 (4%) | 6 (0.4%) | 81 (4%) | 1750 (6%) | 56 (4%) |
|  | TUGs with GO terms | 2018 (57%) | 1208 (86%) | 1308 (62%) | 17980 (66%) | 1010 (75%) |
